# A Thirty-Year Follow-Up Surveillance Study for Neoplasia of a Dutch Ulcerative Colitis Cohort

**DOI:** 10.1155/2013/274715

**Published:** 2013-11-28

**Authors:** J. A. M. Stolwijk, A. M. J. Langers, J. C. Hardwick, R. A. Veenendaal, H. W. Verspaget, R. A. van Hogezand, H. F. Vasen, A. E. van der Meulen-de Jong

**Affiliations:** Gastroenterology Department, Leiden University Medical Center, Postbus 9600, 2300 RC Leiden, The Netherlands

## Abstract

*Background*. Patients with ulcerative colitis have an increased risk of developing colorectal cancer (CRC). The aim of this study is to assess the yield of surveillance colonoscopies in a tertiary referral cohort of ulcerative colitis patients and to identify different risk groups for dysplasia. *Methods*. A cohort of 293 patients (148 males, mean age 33.8 years at diagnosis) was built up at our center and started the surveillance program 8–12 years after start of symptoms. They underwent colonoscopies every one to three years. Endpoints were dysplasia or a (sub)total colectomy. *Results*. After a follow-up period of 10 years, the cumulative incidence of any dysplasia was 23.5%, and of CRC 4.0%. After 15 years these percentages were 33.3% and 6.8%. Patients with pancolitis (*n* = 178) had a significantly higher cumulative risk of dysplasia than patients with distal disease, HR 1.9 (95%CI 1.1–3.3). Patients who started surveillance at an older age are at increased risk for any dysplasia, HR 1.03 (95%CI 1.01–1.05). *Conclusions*. This prospective surveillance study shows a high yield of dysplasia in ulcerative colitis patients. We recommend developing separate surveillance programs for different risk groups. In our opinion patients with distal colitis can follow the general population surveillance program.

## 1. Introduction

Patients with inflammatory bowel disease (IBD) with colon involvement (both Crohn's disease (CD) and ulcerative colitis (UC)) have a 2.4-fold increased overall colorectal cancer (CRC) risk [1]. CRC accounts for 10–15% of all deaths in IBD patients, and IBD-related CRC accounts for 1-2% of all CRC cases in the general population [2, 3]. There is general agreement that the risk of developing CRC is associated with the extent of disease in the colon and duration of disease. Whether young age at onset of disease is an independent risk factor is controversial [4, 5].

In IBD patients with inflammation in at least 30% of the colon, colonoscopic surveillance is recommended [6–9]. Although the effectiveness is limited, it is the best tool available to detect neoplasia at a curative stage and thus lead to a better prognosis [8, 10].

A meta-analysis based on a selection of 19 UC studies performed between 1961 and 1995 reports a cumulative incidences of CRC of 1.6%, 8.3%, and 18.4% after, respectively, 10, 20, and 30 years of disease [4]. In more recent studies, the cumulative incidences were 0%, 2.5%, and 7.6% in a cohort with 600 UC patients [9] and 1.0%, 2.4%, and 4.8% in a follow-up study during 24 years in 4125 UC patients [11]. A meta-analysis from Jess et al., including 8 studies from 1995 to 2009, showed overall cumulative colorectal cancer incidence of <1.0% after 10 years and 1.1%–5.3% after 20 years of follow-up [1].

The decrease in CRC risk seen in more recent studies could be explained by more effective treatment or better colonoscopy techniques to detect abnormalities and enabling endoscopic and surgical intervention before cancer develops [9, 12].

Despite a decrease in UC-associated CRC over time, the diagnosis of CRC is delayed or missed in about 20% of IBD patients even when monitored according to guidelines [6]. Clearly, current UC surveillance practice leaves considerable room for improvement in detecting CRC in an early and curable stage.

The aim of this study is to study the yield of surveillance of the colon in a tertiary referral cohort of UC patients in the period 1980–2012 and to identify high risk groups for dysplasia.

## 2. Methods

In a prospective follow-up study, a cohort of 293 UC patients was built up at our center between 1980 and 2005. All patients were 18 years and older at first surveillance colonoscopy and the diagnosis of UC was endoscopically and histologically confirmed. All patients had an intact colon eight years after the onset of symptoms. The location of the disease was registered according to the Montreal classification [13]. Patients with proctitis were not included. The date of the first surveillance colonoscopy (between 1980 and 2005) served as the entry point of the study. Patients were offered colonoscopies every three years in the second decade of disease, biannually in the third decade, and annually from the fourth decade onwards. Patients with primary sclerosing cholangitis (PSC) were offered annual colonoscopies starting at the time of PSC diagnosis. During surveillance, colonoscopies 4 at random biopsies were taken every 10 cm from coecum to rectum. Extra biopsies were taken if there were local abnormalities and polyps [14]. The follow-up time was till April 1, 2012. Endpoints were dysplasia, loss to follow-up, or a (sub)total colectomy.

The database of the patients included information about sex, age at onset of symptoms, age at diagnosis of UC, age at first surveillance colonoscopy, extent of colitis (distal or pancolitis), dates and findings of the colonoscopies, histology of the colonic biopsies, location and type of dysplasia and stage of cancer [15], surgery, and date of last surveillance endoscopy. The most severe dysplasia found at each colonoscopy was recorded in the database.

For classification of the degree of dysplasia the criteria of Riddell were used (negative for dysplasia, indefinite for dysplasia, low-grade dysplasia, high-grade dysplasia, and invasive cancer) [16].

*Statistical Analysis. *All statistical analyses were performed with SPSS version 17.0. The Kaplan-Meyer survival analysis was used to estimate the follow-up time to event (LGD, HGD, and CRC). Date of last colonoscopy and (sub)total colectomy were censoring events. The Cox regression model was used to predict the influence of different variables on the outcome of surveillance colonoscopies.

## 3. Results

### 3.1. Patient Demographics

A cohort of 293 patients (148 males) was built up at our center. The characteristics of the study cohort are shown in Table 1. Sixty-one percent (178 patients) had pancolitis. In 15 patients (5.1%), PSC was diagnosed. At first surveillance, colonoscopy LGD was diagnosed in 9 patients (3.1%) and none of the patients had HGD or CRC. Dysplasia of any grade was detected in 72 patients (24.6%) during the study period which encompassed 2639 person years of follow-up.

A total of 245 patients underwent 1115 surveillance colonoscopies (mean follow-up period 10.8 (sd. 6.9) years). The mean age at last colonoscopy was 53.1 (sd. 14.8) years; 46 patients were older than 70 years. Dysplasia of any grade was detected in 8.3% (92/1115) of the follow-up colonoscopies; LGD in 6.6% (*n* = 74), HGD in 0.5% (*n* = 6), and CRC in 1.1% (*n* = 12).

39 patients died during follow-up at a mean age of 68.1 years. The causes of death were CRC (*n* = 6), other cancers (*n* = 7), cardiovascular disease (*n* = 7), and other causes (*n* = 19).

**Figure 1:**
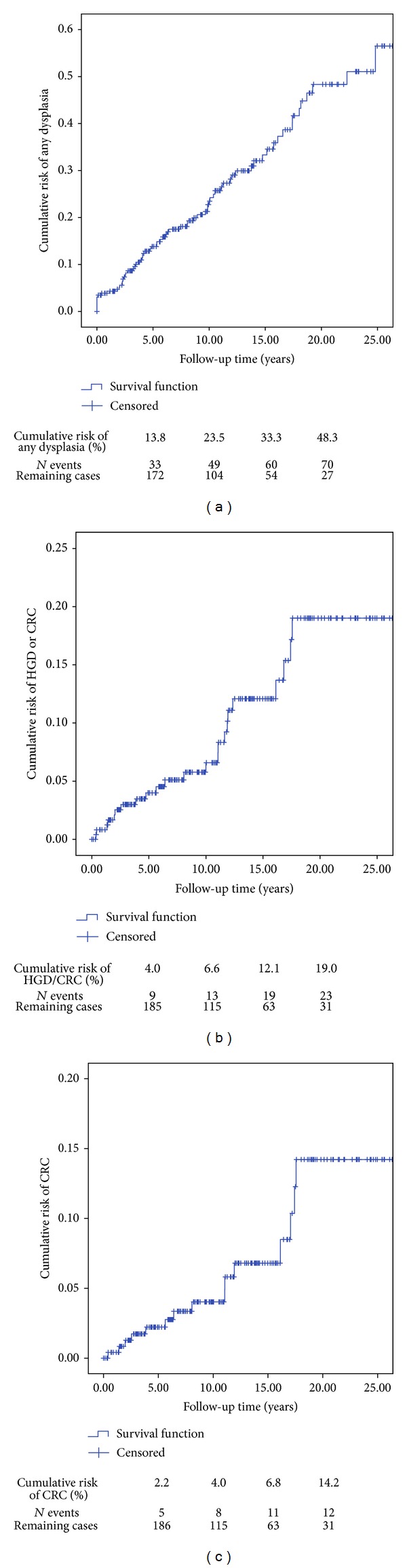
(a) Cumulative risk of any dysplasia. (b) Cumulative risk of HGD and CRC. (c) Cumulative risk of CRC.

After a follow-up period of 10 years, the cumulative incidence of patients with any dysplasia was 23.5%, for HGD 6.6% and for CRC 4.0%. After 15 years these percentages were 33.3%, 12.1%, and 6.8%, respectively (Figures 1(a)–1(c)). In patients with pancolitis the cumulative risk for developing any dysplasia was 15.0% after 5 years, 28.5% after 10 years, and 38.6% after 15 years. For patients with distal colitis these cumulative risks are 11.9%, 13.4%, and 22.5% (Log rank 5.6, *P* = 0.016). The cumulative risk for developing HGD in patients with pancolitis was 5.6% after 5 years, 9.6% after 10 years, and 18.1% after 15 years. For patients with distal colitis this risk was 1.1% after 15 years (just one patient) (Log rank 10.8, *P* = 0.001).

Table 2 shows the number of patients with dysplasia adjusted for sex, extent of disease, and age at first surveillance colonoscopy by the Cox proportional hazard model, univariate, and multivariate analysis. There was no difference in detection rate of dysplasia between males and females. Patients with pancolitis had a significantly higher cumulative risk of developing any dysplasia in comparison with patients with distal UC (HR 1.89, *P* = 0.002). For HGD/CRC, the hazard ratio was 13.51 (*P* = 0.01). The age at first surveillance colonoscopy had a significant contribution in the prediction of any dysplasia. Patients receiving their first surveillance colonoscopy at an older age are at increased risk for any dysplasia of the colon, independent of disease duration, HR 1.03 (*P* = 0.007). For HGD and CRC, this increased risk at older age was not seen. In this cohort, the starting point of surveillance in years from onset of symptoms was not a significant predictor of detection of any dysplasia during the follow-up period (HR 1.01, *P* = 0.78) nor for CRC (HR 1.03, *P* = 0.58).

### 3.2. Carcinogenesis

Low-grade dysplasia was detected in 83 colonoscopies in 55 patients. The next colonoscopy after LGD detection was 27 times within one year and 31 times after 1 year (max. 5.1 years). In 25 cases, no next colonoscopy was done because of loss to follow-up or comorbidity, or a surgical resection procedure (8 cases). Ten patients, 18.2% (10/55), showed progression of LGD to HGD and/or CRC. In eight colonoscopies (7 patients), progression of LGD to HGD and/or CRC was seen in less than 2 years and 3 months after LGD detection. In two other patients, HGD was detected in the resection specimen. In the tenth patient, CRC was found by CT scan 15.2 years after the last surveillance colonoscopy and 16.1 years after LGD detection.

Concerning the HGD/CRC patients, LGD preceded HGD/CRC in 10 of 23 (43.5%) patients. No significant differences in patient characteristics were seen between the patients with and without LGD prior to HGD and/or CRC.

#### 3.2.1. Colorectal Cancer

During the follow-up period, one patient was diagnosed with a neuroendocrine colon tumor and one patient with a colon localization of a B-cell lymphoma. A total of 15 patients (5.1%) were diagnosed with colorectal adenocarcinoma (14/178 patients with pancolitis; 7.9%) during the follow-up period at a mean age of 49.2 years (range 24.9–65.6 years). Two patients were known with PSC. The shortest interval from onset of symptoms of UC till CRC diagnosis was 10.2 years. The mean interval from last surveillance colonoscopy until CRC diagnosis was 2.4 (sd. 3.8) years.

Twelve CRCs were detected during a surveillance colonoscopy. One cancer was diagnosed in a resection specimen; two were detected by a CT scan performed because of abdominal complaints. Three tumors were located in the rectum, six were distal colon tumors, and six were found in the right side of the colon. Eight patients were at stage I (*n* = 3) and stage II (*n* = 5). Stage III was diagnosed in four patients and stage IV in three patients. In 7 patients, CRC was not preceded by surveillance-detected dysplasia. Eight of the 15 CRC patients died, from which 6 due to CRC at a mean age of 49.0 (sd. 14.5) years.

#### 3.2.2. Resection Specimens

Patients who underwent colorectal surgery were censored at the date of last surveillance colonoscopy before surgery. Results from last surveillance colonoscopy and resection specimen were compared. A total of 61 patients underwent a surgical resection procedure, 36 patients because of therapy resistant UC and 25 patients because of any degree of dysplasia. In 50 patients the resection specimen confirmed the outcome of the colonoscopy (including biopsies) performed before the surgery. In 8 patients, the resection specimen showed a higher degree of dysplasia than had been diagnosed at colonoscopy; in 3 patients the degree of dysplasia detected in the surgical specimen was lower than had been reported by the pathologist based on the biopsies taken at colonoscopy.

## 4. Discussion

This large prospective surveillance study in a tertiary referral center among patients with UC shows a low yield of dysplasia at the first life-time surveillance colonoscopy. During follow-up endoscopies, the yield is significant. No difference in sex was observed. Patients with extensive disease have a higher risk of developing dysplasia/CRC. Patients who have their first surveillance colonoscopy at older age are at increased risk for any dysplasia of the colon, independent of disease years. Nearly half of the patients with HGD/CRC were diagnosed with LGD before.

We found any dysplasia in 72 of 293 UC patients (24.6%). The same proportion, 25%, was found in 2005 by Lindberg et al. [17]. This study shows an overall CRC incidence of 5.1% in contrast to other studies with incidences of 1.6–3.7% [1, 4, 11]. We detected a cumulative incidence of any dysplasia of 23.5% by 10 years and 48.3% by 20 years and of CRC these incidences were 4.0% after 10 years and 14.2% after 20 years. Our dysplasia cumulative incidence is quite higher than in most series, with cumulative incidences of 1.5–10.0% for any dysplasia and 0%–8% for CRC [4, 9, 18]. These differences have several possible explanations.

The majority (61%) of the study population had pancolitis, with an increased risk for any dysplasia compared to the patients with distal colitis, HR 1.9 (*P* = 0.02). Patients with pancolitis had a significantly higher incidence of CRC than patients with distal colitis, HR 8.4 (*P* = 0.04). On the other hand, even in patients with pancolitis the incidence of CRC is higher in our cohort compared with previous cohorts (7.9% in this study versus 5.4%) [4, 11]. Another reason for the relatively high incidence of dysplasia in our cohort could be the method of analysis. In previous studies, patients with colectomy remain in the analysed study population, which decreases the yield of the surveillance [4, 6]. Lack of mucosal healing in a substantial proportion of our cohort is probably the major reason for the high incidences of CRC and any dysplasia. Analyzing the fifteen CRC patients, they were not in clinical remission before CRC diagnosis. Despite medication use, chronic active (multi)focal disease and scar tissue were seen at endoscopy and/or in biopsies. The LUMC is a tertiary referral center where mainly severe cases are treated.

In a meta-analysis of Jess et al. a gender-related risk was found, in favor of females, probably related to a protective effect of estrogens in women [1]. We found slightly more CRC in women than in men in this study, though not significant, HR 0.75 (*P* = 0.6).

The mean age at diagnosis of UC in our cohort is the same as the patient populations in previous studies [6, 19, 20]. The median age at CRC diagnosis was 49.9 years (range 24–71) and this is comparable to results from other studies (49.0–55.5 years) [6, 9, 21]. In a study from 2008, the left colon was the main location for CRCs; our study corroborates these findings with 60% of CRCs (9/15) located in the left colon [6].

In a meta-analysis from 2001, a slight increase in the incidence of CRC over time was found, explained by an increased CRC detection by surveillance programs [4]. This is in contrast to the study of Rutter et al. Their results showed a significant reduction in the incidence of CRC over time [9].

The benefit of a surveillance program for patients at increased risk for CRC is not clear from our study. On the one hand, twelve of fifteen (80.0%) patients were diagnosed with CRC at surveillance colonoscopy, which is comparable with previous studies [22]. On the other hand, we found a higher proportion CRCs in stage IV and a lower proportion in stage I at diagnosis in comparison with other studies [22, 23], with proportions more similar to the nonsurveillance population of the study of Lutgens, though we cannot make any conclusion about it because of the small absolute number of CRCs in our cohort [22].

Although the interval between onset of symptoms of UC and first surveillance colonoscopy was not a significant predictor for detection of dysplasia in our study, the earliest cancer 10.2 years, after onset of symptoms, provides further support for starting surveillance 10 years after the onset of symptoms.Despite the high yield of dysplasia of the surveillance program in our study, 20% of CRCs presented as interval cancers between surveillance examinations. Gastroenterologists may overlook dysplasia of the mucosa of the colon and it is important to realise that dysplasia and cancer can still arise despite adherence to a surveillance program [17, 24].

The reported progression rates of LGD to HGD and CRC have a wide range from 7.1% of 28 LGD patients to 39.1% of 46 LGD patients [21, 25]. In our cohort, this rate was 18.2% of 55 patients. In 70% (7/10) of these patients, LGD showed progression in less than two years and three months. This finding would support the current practice of decreasing the interval to the next colonoscopy after LGD detection [14, 26].

Mortality due to CRC has decreased gradually over the last decades [2, 17, 27, 28]. Our patients entered the study in the period 1980–2005. The surveillance strategies were not changed during this period. In 2010, the IBD surveillance program of the LUMC was changed in line with the revised recommendations of the British Society of Gastroenterology for colorectal cancer screening in IBD patients published in 2010 [12, 26]. In the current study, we did not use adjunctive colonoscopy techniques like chromoscopy and NBI. Probably these techniques will further increase the detection rate of dysplasia and decrease the incidence of colorectal cancer.

In summary, this study provides further evidence that patients with pancolitis should enter a surveillance program 10 years after the start of symptoms [12]. This study suggests that patients with left-sided colitis have a similar CRC risk as the general population and do not need a more intense surveillance schedule. This study confirms that a surveillance program is capable of detecting the great majority of CRCs before they become symptomatic and that this may lead to a survival benefit.

## Figures and Tables

**Table 1:** Characteristics of the patient population (*n* = 293).

	Mean (sd)	Range	
	years	Min.	Max.
Age at diagnosis	33.8 (13.8)	4.1	69.4
Age at first surveillance colonoscopy	44.1 (13.8)	18.2	79.3
Age at end of follow-up	53.1 (14.8)	21.2	84.0
Time from onset of symptoms to first surveillance colonoscopy	12.0 (4.7)	8.0	38.0
Follow-up period of intact colon*	10.8 (6.9)	0.1	27.1
Interval between surveillance colonoscopies*	2.0 (1.2)	0.1	10.1
Age at death (*n* = 39)	68.1 (16.3)	26.3	97.6

*Only patients with follow-up colonoscopies, *n* = 245.

**Table:** (a) Positive for any dysplasia

Factors	Number (%)	Univariate			Multivariate		
		HR	95% CI	Sig.	HR	95% CI	Sig.
Sex		1,552	0.963–2.502	0.071	1,430	0.878–2.329	0.150
Male	45/148 (30.4)						
Female	27/145 (18.6)						
Location		1,922	1.115–3.312	0.019	1,918	1.111–3.311	0.019
Pancolitis	55/178 (30.9)						
Distal colitis	17/115 (14.8)						
Age at 1st surveillance, yrs		1,032	1.013–1.051	0.001	1,028	1.009–1.047	0.004

**Table:** (b) Positive for HGD/CRC

Factors	Number (%)	Univariate			Multivariate		
		HR	95% CI	Sig.	HR	95% CI	Sig.
Sex		1,354	0.586–3.129	0.479	1,460	0.624–3.421	0.383
Male	14/148 (9.5)						
Female	9/145 (6.2)						
Location		13,232	1.783–98.182	0.012	13,508	1.818–100.384	0.011
Pancolitis	22/178 (12.4)						
Distal colitis	1/115 (0.9)						
Age at 1st surveillance, yrs		1,008	0.975–1.042	0.654	1,002	0.968–1.036	0.926

**Table:** (c) Positive for CRC

Factors	Number (%)	Univariate			Multivariate		
		HR	95% CI	Sig.	HR	95% CI	Sig.
Sex		0.748	0.271–2.064	0.575	0.874	0.310–2.460	0.798
Male	7/148 (4.7)						
Female	8/145 (5.5)						
Location		8,305	1.092–63.165	0.041	8,402	1.102–64.062	0.040
Pancolitis	14/178 (7.9)						
Distal colitis	1/115 (0.9)						
Age at 1st surveillance, yrs		0.986	0.943–1.031	0.531	0.984	0.941–1.029	0.486

## Abbreviations

CRC: Colorectal cancer
LGD: Low-grade dysplasia
HGD: High-grade dysplasia
UC: Ulcerative colitis
IBD: Inflammatory bowel disease
PSC: Primary sclerosing cholangitis
LUMC: Leiden University Medical Center
HR: Hazard ratio
Cum.Inc.: Cumulative incidence
CI: Confidence interval
Sd.: Standard deviation.
